# Longitudinal surveillance and transmission of *Acinetobacter baumannii* using whole genome sequencing—a tale of two hospitals

**DOI:** 10.1017/ash.2025.10092

**Published:** 2025-08-08

**Authors:** Chetan Jinadatha, Hosoon Choi, Sorabh Dhar, Keith S. Kaye, Munok Hwang, Jing Xu, Thanuri Navarathna, John David Coppin, Piyali Chatterjee

**Affiliations:** 1 Department of Medicine, Central Texas Veterans Health Care System, Temple, TX, USA; 2 Department of Research, Central Texas Veterans Health Care System, Temple, TX, USA; 3 Division of Infectious Diseases, School of Medicine, Wayne State University, Detroit, MI, USA; 4 Department of Internal Medicine, John D Dingell Veterans Affairs Medical Center, Detroit, MI, USA; 5 Department of Medicine, Rutgers Robert Wood Johnson Medical School, New Brunswick, NJ, USA

## Abstract

**Objective:**

*Acinetobacter baumannii* is known to cause global outbreaks and routine surveillance to prevent nosocomial transmission has historically been limited. A longitudinal surveillance study of *Acinetobacter* isolates using whole genome sequencing (WGS) and whole genome multilocus sequence typing (wgMLST) was performed to map the distribution of sequence types (STs) and intrahospital transmission.

**Methods:**

All *Acinetobacter* clinical isolates were collected in two hospitals (H1, H2) from fifteen units between 2017 and 2021 in Southeast Michigan and analyzed. The isolates were subjected to WGS using the NextSeq instrument (Illumina). The contigs were *de novo* assembled using SPAdes (v3.7.1) and wgMLST analysis was performed using BioNumerics software v7.6. Minimum spanning tree (MST) and dendrograms were created to map distribution of STs and putative transmissions.

**Results:**

ST2^Pas^ was the most prevalent in both hospitals (H1:47.2% and H2:59.7%), followed by ST406^Pas^ (H1:11.1%, H2:8%). ST15^Pas^ (H1:9.7%) was only found in H1. Transmission was mapped for ST2^Pas^, ST406^Pas^ (H1, H2), and ST15^Pas^ for H1 and mainly located in the ICU settings.

**Conclusions:**

Presence of several STs (ST2^Pas^, ST406^Pas^, and ST15^Pas^) prevalent from both hospitals suggest that these are common circulating strains in the area. Sporadic transmission events mainly in the ICU settings in both hospitals (H1 and H2) were noted indicating attention to enhanced infection prevention and control measures. Given that *Acinetobacte*r infections are predominantly hospital acquired, an effective surveillance plan incorporating WGS and wgMLST may improve the ability to identify and track trends rapidly, implement effective infection control intervention, and reduce healthcare-associated infections (HAIs).

## Introduction

Gram-negative pathogens such as *Acinetobacter baumannii (A. baumannii)* are often associated with nosocomial outbreaks.^1–3^ The ability of *Acinetobacter* to survive on healthcare surfaces for a prolonged period ^4^ provides an opportunity for these pathogens to be transmitted to another patient if proper infection prevention and control measures are not followed. Moreover, *Acinetobacter* can quickly acquire resistance genes to multiple antibiotics thereby making it increasingly difficult to treat infections with currently available antibiotics.^5^ With the advent of microbial whole genome sequencing (WGS) with high discriminatory power, decreasing cost, and shorter turnaround time, real-time surveillance with the potential for early identification and curtailing larger outbreaks are possible.^6–9^

Healthcare-associated infections (HAIs) due to *Acinetobacter* are known to cause significant morbidity and mortality in patients and poses a major public health threat.^3^ According to the Centers for Disease Control and Prevention, 1 in 31 hospitalized patients and 1 in 43 nursing home residents acquire HAIs. The Centers for Disease Control and Prevention also reported that progress had been achieved in reducing device-related HAI until 2019; however, challenges associated with the COVID-19 pandemic reversed these advancements resulting in an increase in the incidence of several HAI-associated antimicrobial resistant pathogens. Several outbreaks involving *Acinetobacter* have been previously reported.^10–12^ WGS has previously facilitated accurate bacterial identification,^13^ mapped genetic relatedness of bacterial isolates,^14^ enabled intrahospital outbreak studies,^1^ and established possible transmission routes.^15^ However, routine surveillance using WGS has not been widely implemented in healthcare facilities. Genomic sequencing has been used successfully for strain/lineage characterization of SARS-CoV-2 to provide information relevant to an immediate public health threat including severity of disease or transmission events.^16,17^ Likewise, epidemiological changes in HAI-causing bacteria captured by WGS surveillance and related monitoring programs can provide clues not only for any gaps in prevention and control strategies but also to aid in shaping antibiotic stewardship programs, even spurring development of new antibiotics.

In this study, we attempt to address several unanswered questions: (1) What type of *Acinetobacter* strain sequence types (STs) are prevalent in the southeast Michigan area and is there genomic diversity among circulating strains in two separate hospitals in the same region? (2) Can we identify any intrahospital transmission clusters within units? To determine molecular epidemiology of circulating hospital *Acinetobacter* strains in southeast Michigan, we performed WGS and whole genome multilocus sequence typing (wgMLST) analysis to characterize genomic diversity and transmission patterns.

## Methods

### Study setting and approval

All patients who are admitted to two separate, geographically distinct tertiary care hospitals in southeast Michigan between 2017 and 2021 were included in this study. Hospital 1 (H1) is a 383-bed hospital, and hospital 2 (H2) is a 248-bed hospital. The isolates were derived from unique patients and collected from fifteen inpatient units comprising of two medical intensive care units (ICUs), two surgical ICUs, one trauma unit, and remaining from non-ICU medical-surgical units. All patients admitted only to these fifteen study units during the study period were included.^18^ Infections were categorized as HAIs according to Dhar *et al.* ^18^ This study was approved by the IRB Committees at the Central Texas Veterans Health Care System and Wayne State University.

### Strain typing

A total of 162 *A.* ba*umannii* isolates that were collected as part of routine clinical care were shipped to the Central Texas Veterans Health Care System from the hospital microbiology laboratories for further analysis. For this study, five isolates were excluded due to lack of available hospital or unit information. No environmental isolates were part of this study. The clinical isolates were first confirmed with Matrix-Assisted Laser Desorption Ionization Time of Flight Mass Spectrometry (BioMérieux, Marcy-l’Étoile, France) prior to sequencing. Any clinical isolates belonging to other members of the *Acinetobacter calcoaceticus–baumannii* complex (twenty-three), including *Acinetobacter pittii*, *Acinetobacter nosocomialis,* and *Acinetobacter calcoaceticus* were excluded from the study.

### Whole genome sequencing

The clinical isolates were obtained and subjected to DNA extraction using the QIAamp DNA Micro Kit (Qiagen, Hilden, Germany). The quality of DNA was measured by Nanodrop (ThermoFisher, Waltham, MA, USA) and Qubit (Life Technologies, Carlsbad, CA, USA). DNA libraries were prepared using the Nextera DNA Flex Library Prep Kit (Illumina, San Diego, CA, USA) according to manufacturer’s protocol. The whole genome libraries were sequenced using the Illumina NextSeq platform (Illumina, San Diego, CA, USA). A minimum Q score of 30 × for assembled genomes, with a coverage depth of greater than 60 × were included.

### Bioinformatic analysis

*De novo* assembly was performed using the SPAdes version 3.7.1 assembler on the BioNumerics platform (Applied Maths NV, Sint-Martens-Latem, Belgium) using the Bowtie2 mapping algorithm. WgMLST (assembly-free and assembly-based calls) was performed using the calculation engine on the BioNumerics version 7.6 platform.

Assembled isolates were assigned to multilocus sequence typing (MLST) Pasteur or Oxford ST schemes. The *A. baumannii* MLST Pasteur scheme uses seven housekeeping genes: cpn60 (60-KDa chaperonin), fusA (elongation factor EF-G), gltA (citrate synthase), pyrG (CTP synthase), recA (homologous recombination factor), rplB (50S ribosomal protein L2), and rpoB (RNA polymerase subunit B).^19^ While the MLST Oxford scheme uses seven housekeeping genes including gltA (citrate synthase), gyrB (DNA gyrase), gdhB (glutamate dehydrogenase), recA (homologous recombination factor), cpn60 (60-KDa chaperonin), gpi (glucose phosphate isomerase), and rpoD (RNA polymerase subunit D).^20^ A minimum spanning tree (MST) was created with STs to demonstrate the clusters in each hospital (H1, H2), each unit (U1-U15), and each year of infection (2017–2021). The MST Figures were generated using BioNumerics version 8.1 (bioMérieux/Belgium). Whole genome single nucleotide polymorphism (SNP) analysis was also conducted using BioNumerics version 7.6 platform (Applied Maths NV, Sint-Martens-Latem, Belgium).

The most common, genetically related STs in each hospital (H1 or H2) were subsequently compared by location (hospital units U1-U15) and time (2017–2021) to identify potential transmission events (clusters). A dendrogram representing the genetic relatedness of isolates subjected to WGS was created using Unweighted Pair Group Method with Arithmetic Mean (UPGMA) with BioNumerics version 7.6 software platform. Potential clonal transmission was determined to occur where SNP threshold is set at ≤ 10^21^ and isolates were determined to be closely related with SNP cut off at ≤ 2.^22^ *A. baumannii* strain K09-14 with NCBI Reference Sequence # NZ_CP043953.1 was used as a reference. Putatively related isolates are grouped into clusters based on SNP threshold described above and shown in dotted rectangles in the figures. The number at each branch point indicates the SNP distance between the isolates.

## Results

### Distribution of ST types across the hospitals (H1 and H2), units (U1-U15), and years (2017–2021)

WgMLST analysis was performed on a total of 134 unique *A. baumannii* patient isolates, 72 of which were from H1 and 62 were from H2. A total of 13 and 15 different STs from H1 and H2, respectively, were identified with 5 STs being in common between the two hospitals. Other unique STs for H1 and H2 were also observed (Tables 1 and 2). ST2 was the predominant ST for both hospitals (H1:47.2%, 34/72; H2:59.7%, 37/62). Using an Oxford scheme, a substantial genetic heterogeneity within the ST2^Pas^ (ST195^Oxf^, ST208^Oxf^, ST281^Oxf^, ST1701^Oxf^, ST2420^Oxf^, ST1599^Oxf^) was observed. In addition to ST2, ST406 (11.1%, 8/72) and ST15 (9.7%, 7/72) were more prevalent in H1 while ST406 (8%, 5/62) and ST427 (4.8%, 3/62) were prevalent in H2. ST406^Pas^ heterogeneity (ST2768^Oxf^ and ST310^Oxf^) was also observed. Of the total 134 isolates, several isolates (Tables 1 and 2) were not assigned a ST type by either one or both MLST schemes.

**Table 1.** Hospital 1

Origin	Pasteur	Oxford	2017	2018	2019	2020	2021	Total
H1			1					1
	ST2		1					1
		ST208	1					1
H1U1			1	3	8	1	5	18
	N/A			1			1	2
		N/A					1	1
		ST281		1				1
	ST15				1			1
		ST236			1			1
	ST2		1		6	1	2	10
		ST1701			1			1
		ST195			2		2	4
		ST208			3			3
		ST2420				1		1
		ST281	1					1
	ST250						1	1
		ST1739					1	1
	ST36			1				1
		ST296		1				1
	ST406			1	1			2
		ST2768		1	1			2
	ST79						1	1
		N/A					1	1
H1U2			6	5	8		3	22
	N/A			1	1		1	3
		N/A		1	1		1	3
	ST118				1			1
		N/A			1			1
	ST1440				1			1
		ST1393			1			1
	ST15						1	1
		ST236					1	1
	ST2		3	1	3		1	8
		ST1599					1	1
		ST208	2		2			4
		ST281	1		1			2
		ST195		1				1
	ST212		1					1
		N/A	1					1
	ST245			1				1
		N/A		1				1
	ST32				1			1
		ST823			1			1
	ST36			1				1
		ST296		1				1
	ST406		1	1	1			3
		ST2768	1	1	1			3
	ST49		1					1
		ST128	1					1
H1U3			1	6	7	2	5	21
	N/A				1		1	2
		N/A			1		1	2
	ST15			2	1		1	4
		ST236		2	1		1	4
	ST2		1	4	4	2	2	13
		ST208		4	4		1	9
		ST2420				1		1
		ST281	1			1	1	3
	ST406				1			1
		ST2768			1			1
	ST54						1	1
		N/A					1	1
H1U7				2	1			3
	N/A			2				2
		ST2768		2				2
	ST2				1			1
		ST208			1			1
H1U11			1	1	2		2	6
	N/A		1		1		1	3
		N/A	1				1	2
		ST2768			1			1
	ST15						1	1
		ST236					1	1
	ST2				1			1
		ST208			1			1
	ST406			1				1
		ST310		1				1
H1U12				1				1
	ST406			1				1
		ST2768		1				1
Grand total			10	18	26	3	15	72

**Table 2.** Hospital 2

Origin	Pasteur	Oxford	2017	2018	2019	2020	2021	Total
H2U4			3	7	5	4	9	28
	N/A						1	1
		N/A					1	1
	ST109						1	1
		ST1927					1	1
	ST2		2	6	3	2	6	19
		ST1701					1	1
		ST195	2	2				4
		ST208		3	1	2	3	9
		ST2420		1			2	3
		ST281			2			2
	ST406		1	1	1	1		4
		ST2768		1				1
		ST310	1		1	1		3
	ST412				1	1		2
		N/A			1	1		2
	ST93						1	1
		ST1068					1	1
H2U5			2	8	3	4	2	19
	ST15			1			1	2
		ST236		1			1	2
	ST2		2	5	1	3	1	12
		ST1701	1	1		1		3
		ST208	1	1	1	1	1	5
		ST195		3		1		4
	ST250					1		1
		ST1739				1		1
	ST268			1				1
		ST471		1				1
	ST291				1			1
		N/A			1			1
	ST427			1				1
		N/A		1				1
	ST647				1			1
		N/A			1			1
H2U6				3	1	1	2	7
	N/A			1				1
		N/A		1				1
	ST2			1			1	2
		ST208					1	1
		ST195		1				1
	ST32						1	1
		ST823					1	1
	ST406					1		1
		ST310				1		1
	ST427			1	1			2
		–		1				1
		N/A			1			1
H2U8				1		2	2	5
	ST126						1	1
		ST955					1	1
	ST150					1		1
		ST2773				1		1
	ST2			1		1		2
		ST1701				1		1
		ST208		1				1
	ST78						1	1
		ST944					1	1
H2U10				1				1
	ST2			1				1
		ST208		1				1
H2U14				1				1
	N/A			1				1
		N/A		1				1
H2U15			1					1
	ST2		1					1
		ST281	1					1
Grand total			6	21	9	11	15	62

Tables 1 and 2: Summarizes the different STs (both Pasteur and Oxford in separate columns and separated by a row) found in different units (H1: Table 1 and H2: Table 2) for each year (2017–2021).

**Figure 1.**
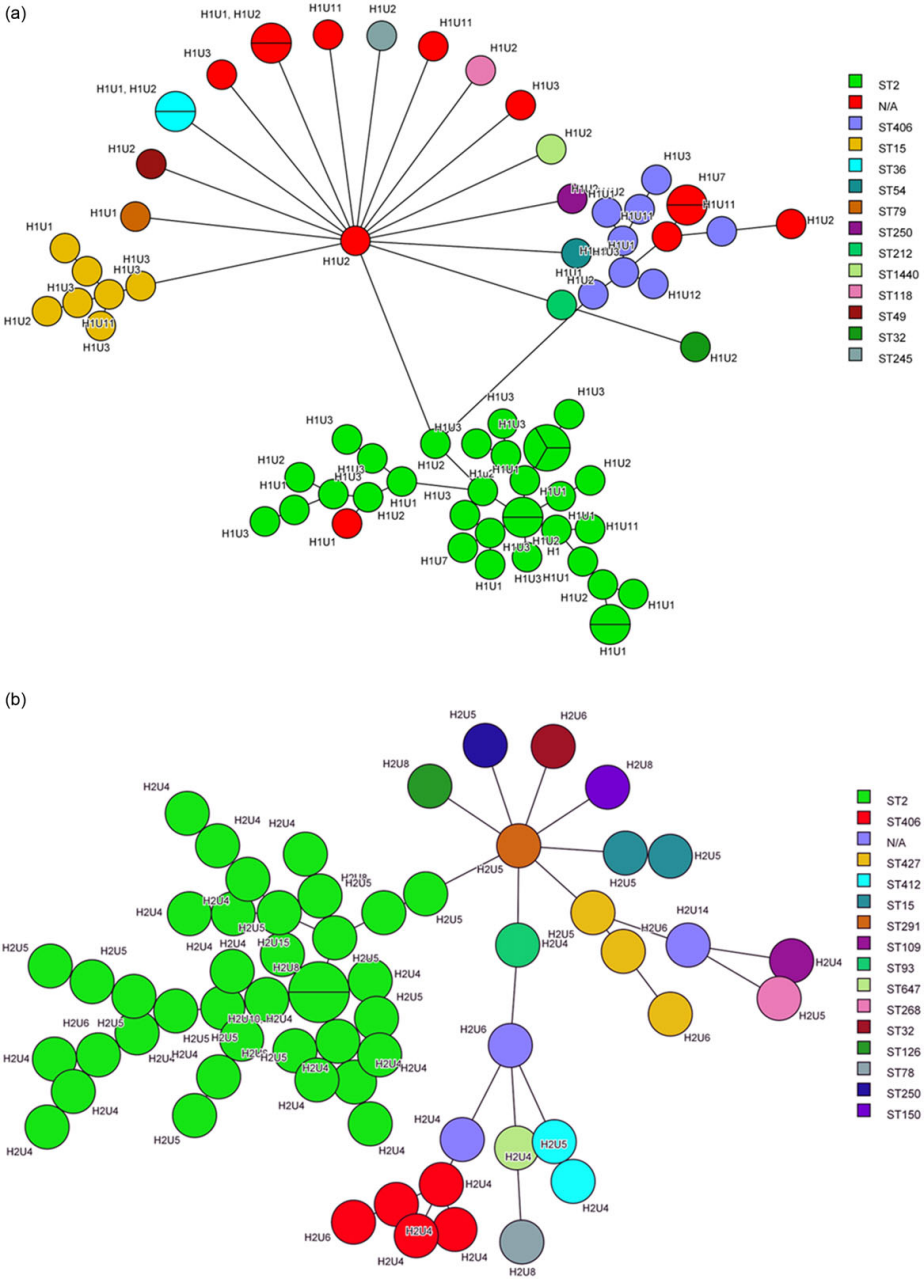
(a, b) Minimum spanning tree of *A. baumannii* clinical isolates in hospitals (H1: Figure a and H2: Figure b) and different wards (U1-U15). For identical strains circles are marked with dividing lines. Each ST is marked on the side bar with different colors.

**Figure 2.**
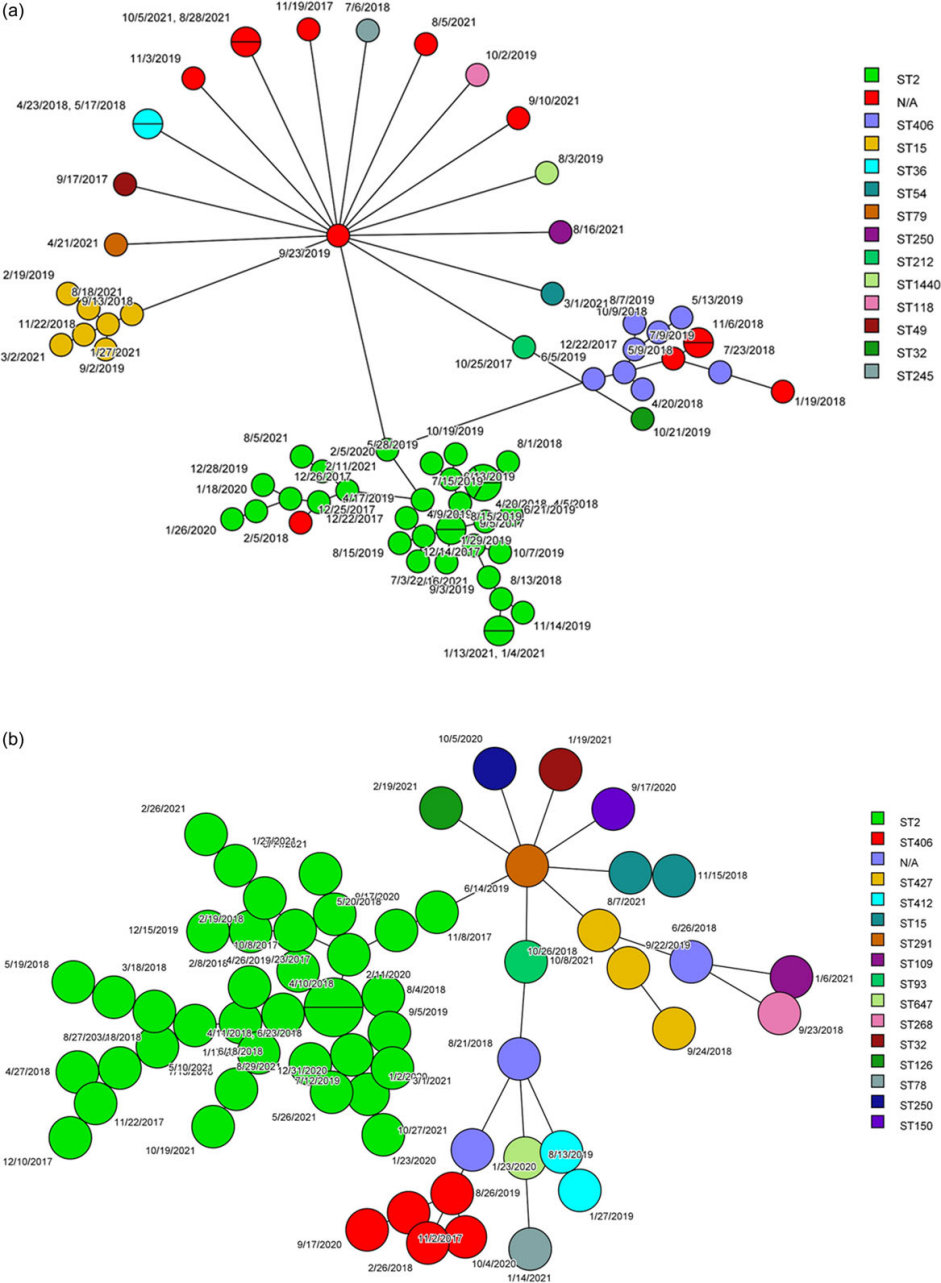
(a, b) Minimum spanning tree of *A. baumannii* clinical isolates in hospitals (H1: Figure a and H2: Figure b) and different years (2017–2021). For identical strains circles are marked with dividing lines. Each ST is marked on the side bar with different colors.

Three units in H1 (H1U1, H1U2, and H1U3) had the highest number and most diverse STs. H1U2 had ten different STs, followed by H1U1 with six STs. Similarly, for H2 two units, H2U4 (43.5%, 27/62) and H2U5 (30.6%,19/62), had the highest number and diverse STs. H2U5 with seven different STs was the most diverse unit. Other units in H2 (H2U6, and H2U8) had four different ST types (Figure 1a,b). A common theme emerged from both hospitals where most of the isolates were found to be in a few units. For H1 and H2, most isolates were predominantly clustered in the ICU setting, while non-ICU units had both fewer number and less diversity of STs. The highest number of isolates collected for H1 were in the year 2019 at 36% (26/72) followed by 2018 at 25% (18/72) that of H2 were in 2018 at 33.9% (21/62) followed by 2021 at 24.2% (15/62). For the year 2018, both H1 and H2 had high rates of *A. baumannii* infection (Figure 2a,b).

### Transmission clusters in two hospitals, H1 and H2

**Figure 3.**
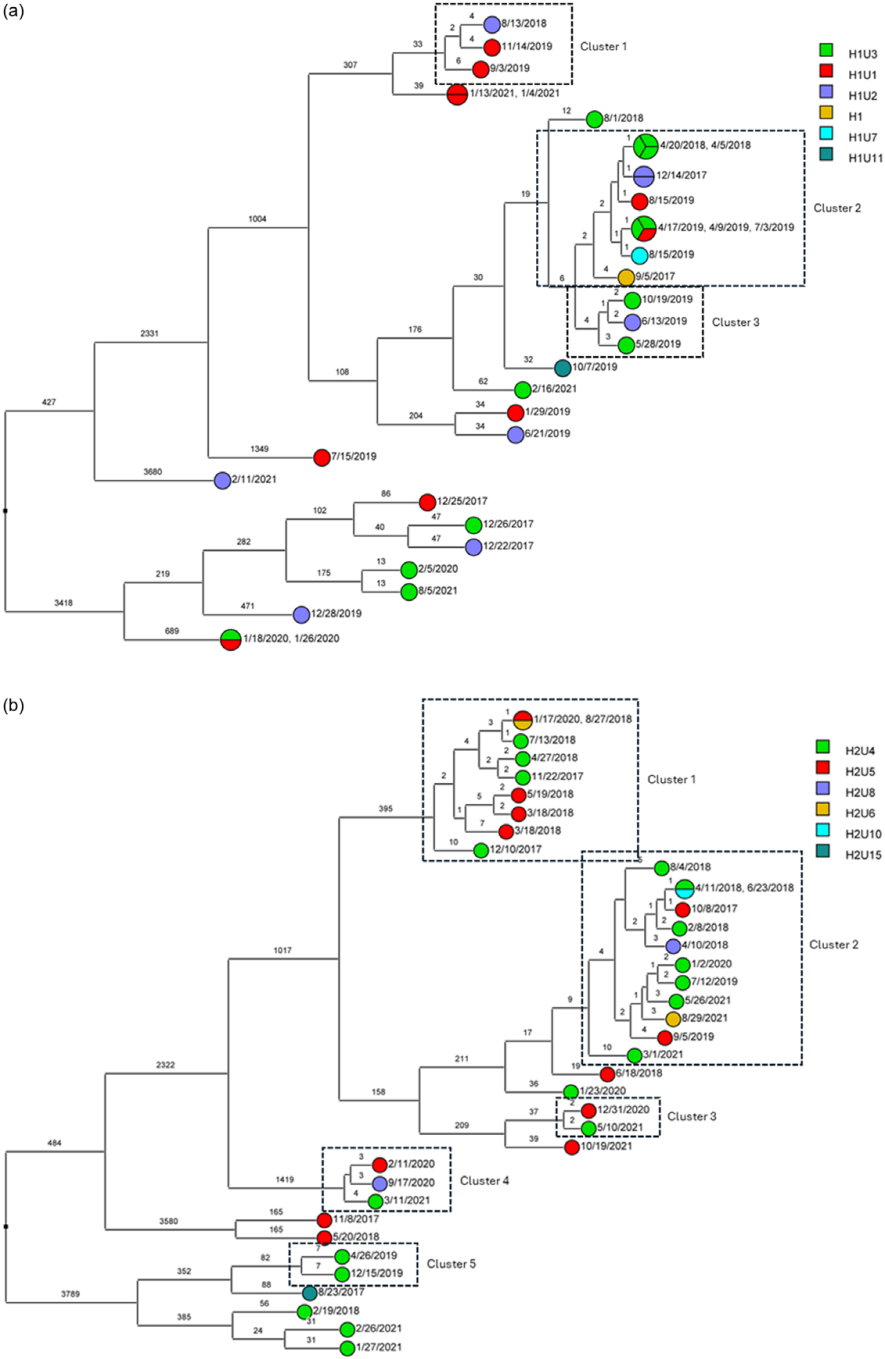
(a, b) Dendrogram demonstrating the SNP differences between ST2^Pas^ sequences of *A. baumannii* clinical isolates in hospitals (H1: Figure a and H2: Figure b) that were related (≤2 SNP differences) or putatively related (≤10 SNP differences) and transmission clusters are marked in dotted rectangles. Each unit is marked on the side bar with different colors.

**Figure 4.**
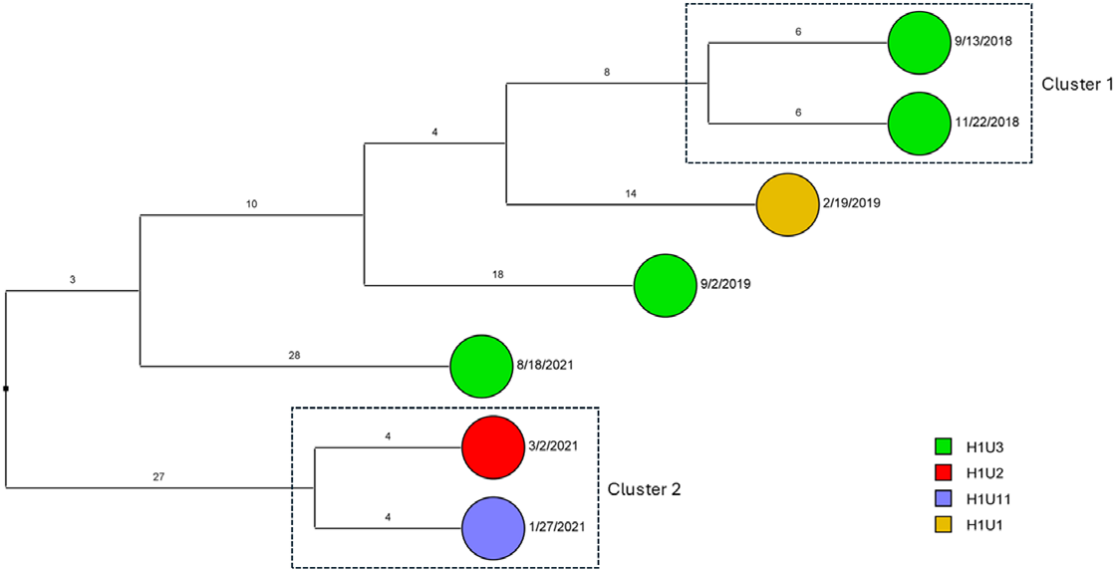
Dendrogram demonstrating the SNP differences between ST15^Pas^ *A. baumannii* clinical isolates in hospitals (H1) that were related (≤2 SNP differences) or putatively related (≤10 SNP differences) and transmission clusters are marked in dotted rectangles. Each unit is marked on the side bar with different colors.

**Figure 5.**
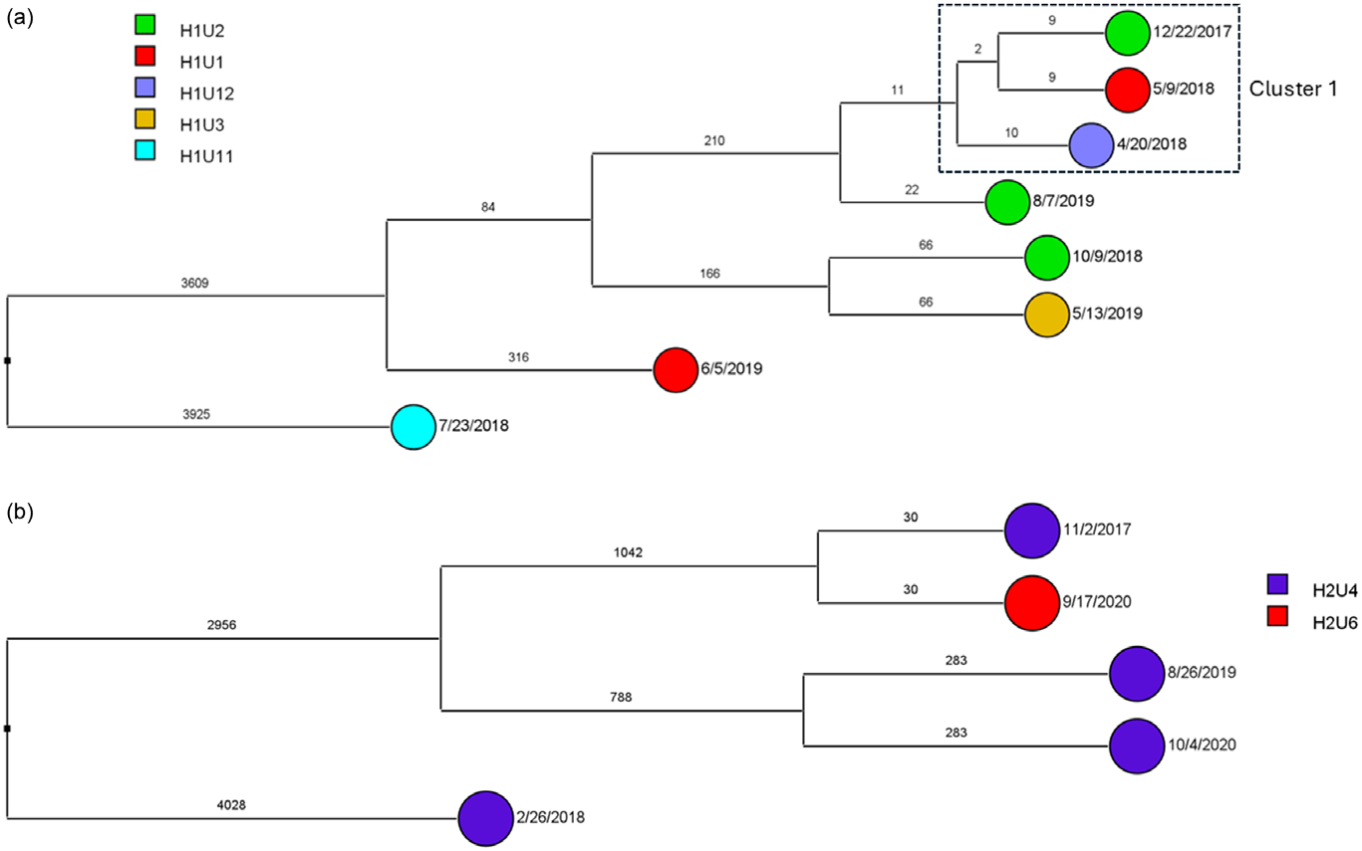
(a, b) Dendrogram demonstrating the SNP differences between ST406^Pas^ *A. baumannii* clinical isolates in hospitals (H1: Figure a and H2: Figure b) that were related (≤2 SNP differences) or putatively related (≤10 SNP differences) and transmission clusters are marked in dotted rectangles. Each unit is marked on the side bar with different colors.

To determine the sequence relatedness among *A. baumannii* isolates with the highest possible resolution, SNPs were utilized. Based on SNPs, there were three transmission clusters for ST2^Pas^ in H1. Two small clusters (Cluster 1 and Cluster 3) were comprised of three isolates (separated by 2–6 SNPs). Cluster 2 was the largest cluster with eleven isolates having three identical pairs (zero SNP difference), and mostly separated by one SNP. Patients may have shared the same units (H1U1, H1U2, H1U3) or different units (H1U7) in proximity. While these clusters were mostly reported in 2019, few were observed in 2017 and 2018 (Figure 3a). For H2, two large clusters were noted along with three smaller putative transmission clusters based on SNP cut off (Figure 3b). These were largely in 2017 and 2018 unlike 2019 reported for H1. The units mostly affected include H2U4 and H2U5. However, some patients from other units such as H2U6, H2U8, and H2U10 also acquired these isolates. For H1, sporadic transmission of ST15 ^Pas^ occurred between patients in unit H1U3 in 2018 and units H1U2 in 2021 (Figure 4). No isolates were recovered from H2 belonging to ST15 ^Pas^. A small sporadic event involving three isolates (9 and 10 SNPs apart) of ST406 ^Pas^ in H1 was noted but none in H2 (Figure 5a,b). The isolates were spread between 2017 and 2018 in three different wards U1, U2, and U12.

Several identical STs were 0 SNPs apart, belonging to ST2^Pas^ (five pairs identical in H1 and two pairs identical in H2) (Figure 3a,b), and ST36 ^Pas^ (two identical in H1) were also observed from different patients in both hospitals (Figure 1a,b).

## Discussion

WGS and epidemiological data combined can trace transmission routes and facilitate rapid intervention by infection prevention and control teams.^23,24^ *In silico* MLST analysis efforts from WGS data have provided better resolution of STs and highlight ST variation among isolates.^25^ Our data suggests considerable heterogeneity of different STs in the southeast Michigan areas, however, ST2 ^Pas^ is the predominant ST circulating in both the hospitals and is mostly endemic to the ICU. We detected ST2^Pas^/ST195^Oxf^, which has previously been reported only in Asia^26,27^ but recently was reported within the USA.^28^ In the present study, we did not identify any ST2^Pas^/122^Oxf^ reported earlier from the Midwest but did find ST2^Pas^/ST208^Oxf^ isolates at the study site. Our data also suggest presence of ST2^Pas^/281^Oxf^ confirming previous reports from Cleveland and Pittsburgh. ST2^Pas^/208^Oxf^ may have shifted toward ST2^Pas^/281^Oxf^.^29^ A PubMLST search (search date until Dec 2024) did find sporadic reports of ST2^Pas^/ST1701^Oxf^ and ST2^Pas^/ST2420^Oxf^ but did not retrieve any ST2^Pas^/ST1599 ^Oxf^ within the USA (two isolates were reported earlier from South Korea and Russia). ST2 ^Pas^ has been known to be extensively drug resistant and reported to be the cause of several outbreaks.^11,12^ ST15 ^Pas^ was reported mostly from Brazil and several European countries and a few from the USA. ST406 ^Pas^ was reported to be circulating within the USA from 2008.

In H1, while several STs were reported from the USA earlier according to PubMLST,^30^ some STs (ST36^Pas^, ST245^Pas^, ST212^Pas^, ST1440^Pas^, ST49^Pas^, ST118^Pas^) were reported to be circulating in other countries such as Czech Republic, China, Japan, Korea, Jordan, and Haiti. In H2, only two STs (ST291^Pas^, ST268^Pas^) were not reported earlier from the USA but were from countries like Lebanon, Spain, and China. Continuous nationwide surveillance data were not captured making it difficult to distinguish between the absence versus lack of circulating above-mentioned STs within the USA.

Our data indicates that multiple sporadic unit level intrahospital transmission occurred during the 5-year span of the study in both hospitals. Although episodes of transmission were found mostly in the ICU settings, other units with high-risk patients or non-ICU units were also affected. Interestingly, the cluster and SNP analysis indicated that the *A. baumannii* isolates may be endemic in such settings leading to acquisition of the infection by a subsequent patient admitted in the same unit and increase the risk of acquisition in other units. Based on our data, infection prevention and control efforts should be mostly geared toward ICUs, but careful consideration should be given to prevent transmission to other high-risk patients such as in neurotrauma unit and non-ICU settings. The in-hospital spread of *Acinetobacter* STs from one patient to the other, raises concerns about infection control practices, the role of environment, transfer of patient between wards or other human factors in the spread of these infections. The two hospitals are separate with differing patients and different staff with no patient overlap or transfers between them. Therefore, inter-hospital transmission was not considered in our study. Surveillance studies using WGS can detect these endemic strains within the hospital and possible transmission routes. This may be crucial in preventing HAIs in the future.

Current limitations of the study include that due to stringent quality requirements of our clinical isolates with the bioinformatic software used, we may have excluded some putative transmission events. The present study was designed only to collect isolates from patients with HAIs. Whether these patients were colonized prior to admission which may have inaccurately contributed to acquisition of HAIs and followed by transmission is unknown. There are currently no commonly utilized prevention bundles that prescreens patients admitted to the hospitals with *A. baumannii* colonization or isolation procedures followed prior to detection of infection. The study was not designed for outbreak tracking in real-time using WGS for infection control practices in the facilities. Standard infection control measures were implemented in each hospital including following contact precautions when an *Acinetobacter* infection is determined.

Our study was not designed to provide a direct comparison between patient-derived isolates with the environmental isolates in and around the patient room simultaneously that may provide clues to the source of transmission leading to infection.

Future studies on identifying the source of these infections are important, including the role of environment (sinks, shared portable equipment, invasive medical devices) or healthcare worker hands. Another interesting area of future study would be to delineate antibiotic resistance patterns of these isolates, if they harbor same types of mutations in the resistance genes and/or carry similar plasmids with new antibiotic resistance genes. In addition, finding factors that allowed these isolates to persist in a hospital setting and promote transmission is crucial. These findings taken together may allow us to develop effective infection control measures to curb and prevent the spread of *A. baumannii*.

In conclusion, we report several circulating ST types, with ST2^Pas^ as the most predominant strain type within the ICU of both hospitals. Environmental hygiene practices as well as infection prevention and control practices geared toward preventing transmission using WGS is possible.
